# Reliable Multihop Broadcast Protocol with a Low-Overhead Link Quality Assessment for ITS Based on VANETs in Highway Scenarios

**DOI:** 10.1155/2014/359636

**Published:** 2014-07-15

**Authors:** Alejandro Galaviz-Mosqueda, Salvador Villarreal-Reyes, Hiram Galeana-Zapién, Javier Rubio-Loyola, David H. Covarrubias-Rosales

**Affiliations:** ^1^Information Technology Laboratory, Center for Research and Advanced Studies (Cinvestav), Science & Technology Park TECNOTAM, Km. 5.5 Cd. Victoria-Soto La Marina Highway, 87130 Ciudad Victoria, TAMPS, Mexico; ^2^Center for Scientific Research and Higher Education at Ensenada (CICESE), 22860 Ensenada, BC, Mexico

## Abstract

Vehicular ad hoc networks (VANETs) have been identified as a key technology to enable intelligent transport systems (ITS), which are aimed to radically improve the safety, comfort, and greenness of the vehicles in the road. However, in order to fully exploit VANETs potential, several issues must be addressed. Because of the high dynamic of VANETs and the impairments in the wireless channel, one key issue arising when working with VANETs is the multihop dissemination of broadcast packets for safety and infotainment applications. In this paper a reliable low-overhead multihop broadcast (RLMB) protocol is proposed to address the well-known broadcast storm problem. The proposed RLMB takes advantage of the hello messages exchanged between the vehicles and it processes such information to intelligently select a relay set and reduce the redundant broadcast. Additionally, to reduce the hello messages rate dependency, RLMB uses a point-to-zone link evaluation approach. RLMB performance is compared with one of the leading multihop broadcast protocols existing to date. Performance metrics show that our RLMB solution outperforms the leading protocol in terms of important metrics such as packet dissemination ratio, overhead, and delay.

## 1. Introduction

Intelligent transport systems (ITSs) aim to integrate information and communication technologies with transportation systems to make transport more efficient, green, safe, and seamless. The ITSs rely on wireless technologies to achieve both vehicle-to-infrastructure (V2I) and vehicle-to-vehicle (V2V) communications. In V2I communications, vehicles communicate with a fixed infrastructure via the wireless media. On the other hand, in the V2V approach vehicles are equipped with wireless communications solutions to directly communicate with vehicles nearby without the need for any infrastructure. The vehicles with V2V capabilities form an ad hoc network, commonly referred as vehicular ad hoc network (VANET). Therefore, in V2V each vehicle must be able to send, receive, and relay safety or infotainment information throughout the VANET. When compared to V2I networks, VANETs provide ubiquitous information sharing and their use results in lower implementation costs, as they work without fixed access network nodes.

The research, development, and standardization communities have identified V2V communications as a key technology to radically improve road safety conditions. It is argued that V2V communications can potentially address above 79% of precrash scenarios involving unimpaired drivers [1]. Regarding nonsafety related applications, it is expected that V2V communications will allow a rapid development and deployment of infotainment applications such as multimedia streaming [2, 3], Internet access [4], and additional infotainment services (e.g., taxi service [5]).

Before VANET technology can fulfill all its expected potential, several difficulties must be addressed. Particularly, the design of methods to effectively disseminate messages through multiple hops is of paramount importance to successfully deploy both safety and nonsafety applications for ITSs [6–8]. For instance, road safety applications attempt to increase the awareness range of drivers by transmitting messages from vehicles internal sensors (e.g., speed) and about surrounding conditions (e.g., crash scenario). These messages are broadcasted to all vehicles located within a specific geographic region or zone of relevance (ZOR). For example, a ZOR can be defined by the lanes of vehicles travelling towards the crash site. From a network perspective, for this scenario the use of broadcast packets to disseminate the messages is more suitable than the use of unicast packets, as the message is of general interest. Additionally, the message must be delivered as soon as possible to all vehicles within the ZOR, such that preventive measures can be timely implemented. In this kind of pre/postcrash warning scenarios, a multihop broadcast (MB) protocol is needed to reach all vehicles within the ZOR at the lowest delivery time.

The design of MB protocols is a challenging task because of spatial-temporal changes in the wireless channel (e.g., fading), different mobility patterns followed by the vehicles, the density of vehicles, and infrastructure availability. In turn, these conditions are closely related with the specific deployment scenario of VANETs [9], the urban and highway scenarios being the most relevant. It is important to note that the constraints imposed by these scenarios are different. Therefore, a MB protocol designed for urban scenarios might not be able to cope with the higher speeds of vehicles in highway scenarios. Furthermore, ITSs implementation in urban scenarios is more likely to rely on V2I solutions, which makes the protocol design more tractable. On the other hand, V2V solutions are the most attractive technology for ITSs deployments in highway scenarios from the cost-efficiency point of view [10]. Nevertheless, the design of MB protocols for these deployments becomes a challenge because of the constraints imposed by highway scenarios. It is noteworthy that highways account for a significant amount of the road infrastructure deployed throughout several countries. For example, highways represent about 75% of the total statute miles in the US [11]. Hence, the design of MB protocols for highway scenarios is an important issue that must be addressed as recently research has pointed out [11–13].

Different wireless technologies have been considered to enable V2V communications (e.g., RFID, IEEE 802.11b, IEEE 802.15.4, Bluetooth, etc.). However, nowadays the most prominent option is the IEEE 802.11p standard [14, 15]. The IEEE 802.11p PHY layer is based on the IEEE 802.11a standard. Similarly, its MAC layer uses carrier sense multiple access with collision avoidance (CSMA/CA). The well-known enhanced distributed channel access (EDCA) mechanism defined in the IEEE 802.11e standard is included in IEEE 802.11p to provide four different access priorities: background, best effort, video, and voice, [14]. However, the IEEE 802.11p standard leaves open the design of efficient broadcast protocols and the solution of issues like the broadcast storm problem (BSP) [7].

The simplest protocol for MB is basic flooding, where each node that receives a packet for the first time retransmits it with no further restrictions. When using CSMA/CA the dissemination of packets by flooding can introduce an important number of redundant broadcasts. This is because of the shared wireless medium nature in CSMA/CA and the lack of any protection mechanism for the broadcast packets. This results in the BSP, where an increase in the medium access delay and in the number of collisions is observed. The BSP is prevalent in networks with high node densities, like those found in vehicular scenarios. The BSP has a negative impact on the arrival time of packets and it can even lead to a significant packet loss.

In order to solve the BSP, beaconless protocols for VANETs aiming at reducing the number of redundant broadcasts have been proposed in the literature [7, 16–18]. Unfortunately, these protocols are not efficient while trying to provide a good trade-off between overhead and reliability [19]. Beacon-assisted protocols that use the neighbors' information to reduce the redundant broadcast have been proposed as well for VANETs scenarios [6, 13, 19, 20]. Although beacon-assisted protocols have shown better performance than beaconless protocols, the accuracy of the information used to make a rebroadcast decision is highly dependent on the frequency of the beacon messages. Even though the overhead/reliability trade-off is better addressed in beacon-assisted protocols, the overhead introduced by the beacon messages can significantly affect the protocol performance.

This paper introduces a new reliable low-overhead multihop broadcast (RLMB) protocol with a cooperative link quality assessment for VANETs in highway scenarios. The proposed RLMB solution provides a good trade-off between overhead and reliability. This is addressed through a beacon-assisted approach along with an implicit acknowledgement mechanism and a position prediction algorithm.

The rest of the paper is structured as follows.  Section 2 discusses the approaches taken by previous studies, while the details of the proposed RLMB protocol are presented in Section 3. The performance of the proposed solution is presented in Section 4, and the concluding remarks are presented in Section 5.

## 2. Related Work

**Figure 1:**
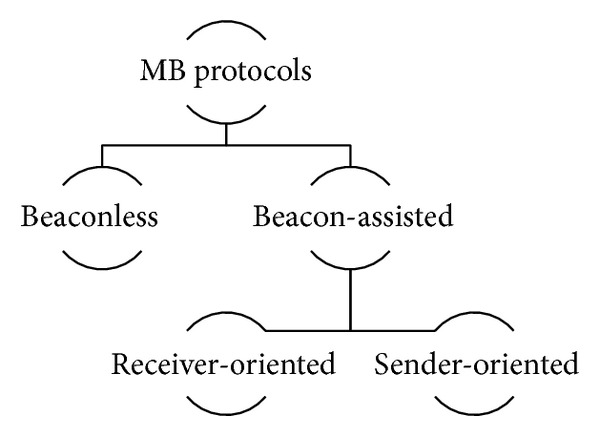
A classification of multihop broadcast protocols for VANETs.

As previously mentioned, multihop broadcast of packets in VANETs was initially addressed with simple flooding, resulting in the BSP. In order to solve the BSP problem, several broadcast storm mitigation protocols for vehicular scenarios have been proposed [12, 16, 19, 20]. In order to discuss different broadcast protocols previously proposed in the literature, the MB protocols will be classified into two main groups within this paper: beaconless (BL) and beacon-assisted (BA) protocols (see Figure 1). A brief explanation of these two groups is provided hereafter.

Basically, BL protocols only use information contained in the disseminated message to decide whether to retransmit it. On the other hand, BA protocols take advantage of the beacon messages that each node in the network transmits periodically. In addition, BA protocols can be further classified as sender-oriented or receiver-oriented. In the latter ones the rebroadcast decision is made at each node when the message is received. Contrastingly, in BA sender-oriented protocols, the next set of rebroadcast nodes is chosen a priori at the previous transmitter node. In both BL and BA protocols, relay decisions can be made based on operational parameters like received power, distance, density of neighbors, timer, or some combination of the former parameters.

There are several BL protocols reported in the literature [7, 16, 17]. In these protocols, each node determines whether to retransmit a message based only on the information contained in the disseminated message. In BL protocols the redundant broadcasts cannot be entirely eliminated, especially under the high variability of scenario conditions present in VANETs. Thus, in BL protocols the trade-off between overhead and reliability cannot be properly addressed.

In BA receiver-oriented (BARO) protocols the exchanged beacons are used for detection of different scenario conditions (e.g., vehicles density). If a current scenario condition reaches a predefined state (e.g., number of transmissions heard, number of neighbors found), then the message is disseminated with a BL approach. Otherwise, a strategy of store-carry-forward is applied (e.g., [12, 20, 21]). Hence as in BL protocols, the trade-off between overhead and reliability cannot be properly addressed in BARO protocols. Therefore, BL and BARO protocols are not suitable for applications with requirements such as high reliability and low overhead.

In the context of BA sender-oriented protocols, the set of relay nodes is formed a priori in the transmitter. The set is chosen based on the stored information gathered through the exchange of hello messages between the vehicles. Thus, given that each node has neighborhood information, the BA sender-oriented approach can potentially reduce the redundant broadcasts in a more efficient way than BL or BARO protocols.

In [22], the enhanced multipoint relay (EMPR) is proposed. This protocol considers the mobility of nodes and an additional area of coverage to select the set of relay nodes. In [23], BA sender-oriented protocol called BPAB is introduced. BPAB performs a repetitive 2-partition method to divide the area inside transmission range. Then, a vehicle to retransmit the message is chosen in the furthest segments. Nevertheless, EMPR and BPAB do not consider the fading nature of the wireless channel when selecting the set of relay nodes. Because of the time-varying channel conditions in V2V communications, the link quality between vehicles could be significantly degraded. Thus, high levels of packet losses and/or delays can occur, as the fading nature of the wireless channel was not considered in the protocol design. Additionally, because of the multipath components, messages beyond the vehicles nominal radio range (NRR) can be occasionally received. As such, these nodes could be considered when selecting the next relay, which would lead to wrong decisions with the consequent waste of resources.

The work presented in [13] introduces a BA sender-oriented protocol, whose aim is to group its neighbor vehicles in clusters formed through the periodic exchange of hello messages. Then, a message is disseminated cluster-to-cluster through the formed transient clustering infrastructure. The proposed cluster formation algorithm and the next relay selection criterion only consider the vehicles mobility. Thus neither of them considered the impairments of the wireless channel in its design, assuming ideal channel conditions. In highway scenarios such assumption can turn into packet losses and/or delays, because it may be difficult to form clusters or an excessive number of clusters may be formed (consisting of a single node), depending on the particular channel conditions at any given moment. Additionally, selecting the next relay only based on vehicles mobility, without observing channel conditions, may also lead to significant packet losses in the presence of a highway V2V channel.

BR-NB and FUZZBR are two sender-oriented MB protocols proposed in [6, 19], respectively. In these two works neighbors are ranked using a fuzzy inference system based on three parameters, namely, vehicle mobility, intervehicle distance, and link quality. Then, the node with the highest rank among its neighbors in the message propagation direction is selected as the next relay. The link quality between two neighbors is estimated in [6] using the hello reception ratio (HRR). A fixed frequency of hello messages is assumed when computing the link quality. Thus for low HRR situations the BR-NB protocol could lead to either transmissions of hello messages with unnecessary high frequency in dense scenarios or to information losses in more dynamic scenarios. Furthermore, the HRR described in [6] is updated for each 10 seconds interval. Thus the reliability of the protocol is likely to be low in high mobility scenarios. For instance, in a highway scenario, vehicles traveling in opposite directions with a typical highway speed (e.g., 32 m/s) can easily go out of range from each other within the assumed 10 seconds period. Additionally, BR-NB needs the 2-hop neighborhood information to estimate both the intervehicular distance and the mobility of vehicles. However, using 2-hop neighborhood information in scenarios with high number of neighbors could cause an exponential growth of the overhead generated by hello packets.

The FUZZBR protocol [19] uses position information of neighbors contained in the hello messages to estimate both intervehicle distances and mobility of neighbors. Because of the predefined mobility patterns in VANETS, using position information is a suitable feature for highway scenarios, as most of the times the same set of vehicles could be used to forward information [9]. Additionally, the FUZZBR protocol described in [19] computes the link quality between two nodes based on the received signal strength indicator (RSSI). Furthermore, the calculation of the vehicle mobility, intervehicle distance, and link quality parameters in FUZZBR is highly dependent on the frequency of hello messages. A consequence of this dependence is that the trade-off between overhead and relevance of the information cannot be entirely addressed. Despite this weakness, FUZZBR is able to adapt to different scenario conditions and overcome several of the problems shown by the MB protocols previously mentioned.

Most of the BA sender-oriented protocols described earlier consider a point-to-point approach for the evaluation of its neighbors. That is, a node establishes the “relay node” suitability of each neighbor using the hello messages broadcasted by the neighbor itself. Hence, in this approach the accuracy of the stored information and the quality of the relay node selection are directly proportional to the hello messages rate (HMR). In conclusion, this approach makes the protocol performance highly dependent on the HMR.

Including the wireless channel impairments in the relay node selection is a challenging and critical issue, as acknowledged by FUZZBR and BR-NB protocols. This issue is studied as well in [24], where the most forward within adjusted radius (MFWAR) mechanism is proposed to adjust the NRR of vehicles in highway scenarios (however note that MFWAR was designed as a discovery service for a unicast routing protocol and cannot be directly compared with a broadcast protocol). Thus, when the point-to-point approach is used for link quality computations in highway scenarios, the needed HMR should be enough to address the time varying nature of the channel. Setting the HMR too high can lead to a larger number of collisions. In contrast, setting a relatively low HMR can lead to not having enough information to perform a proper decision. Hence, with a point-to-point approach BA sender-oriented protocols may achieve a good performance in terms of the existing trade-off between redundant broadcasts and reliability. However, a relevant issue is that the existing trade-off between overhead and the relevance of the information was not addressed.

Based on the previous discussion, it can be stated that a significant issue that must be addressed when designing a new MB protocol for VANETs in highway scenarios is the design of a reliable algorithm for selecting the set of relay nodes while reducing the dependence of the MB protocols from the HMR. As explained in Section 3, the protocol introduced in this paper addresses these design constraints.

## 3. Proposed RLMB Protocol

This paper introduces a new reliable low-overhead multihop broadcast (RLMB) protocol. The development of RLMB considered the inherent constraints of the wireless channels and the high dynamics of vehicles in VANETs for highway scenarios.

RLMB is a BA sender-oriented protocol, thus a subset of relay nodes is selected a priori among the neighbors of the current relay. Then, the selected subset of relay nodes is attached to the message header before the current relay retransmits the message. When receiving a message each node will retransmit it if its own ID is in the header; otherwise the message is dropped. Additionally, each node periodically broadcasts its transmission power, geographic position, and speed in the hello messages.

When selecting the relay set, RLMB proposes the use of a dynamic factor (*β*_*rd*⁡_) to adjust the nominal radio range (NRR) according to the vehicles relative direction (*rd*⁡). Then, the farthest neighbor in each message direction within the adjusted radio range is included in the relay set. The main objective of the *β*_*rd*⁡_ factor is to adjust the NRR to the larger distance where a stable communication can be achieved, this considering the current conditions of the deployment scenario. For this purpose, RLMB uses the signal strength attenuation of the received* hello* messages in each *rd*⁡ and the receiver-transmitter distance for *β*_*rd*⁡_ computation.

It is expected that while the transmitter-receiver distance increases the signal strength attenuation increases as well. However, depending on the scenario channel conditions (e.g., shadowing, fading or interference), a larger or smaller signal attenuation than the expected may occur. Thus, an adaptive *β*_*rd*⁡_ computation is needed in dynamic scenarios. As such, RLMB uses a smart and adaptive fuzzy inference system for the radio range adjustment.

RLMB also uses a position prediction (PP) algorithm. The goal of this algorithm is to fill the gap between the last values stored in the neighborhood table and the most up-to-date values. Additionally, an implicit acknowledgment (iACK) mechanism is coupled to RLMB in order increase its reliability. The iACK mechanism is used when the current relay does not overhear the retransmitted message from one or more of its neighbors in the relay set.

The different mechanisms of the RLMB protocol are explained in the following subsections.

### 3.1. *β*_*rd*⁡_ Radio Adjustment Calculation

The coverage range of each radio transceiver depends on radio channel impairments, which in turn are affected by specific characteristics such as density of nodes, weather, and buildings. Therefore, it can be inferred that considering the transmitter NRR coverage for the selection process may not be the best option, since the channel impairments may be the cause of significant packet losses near the border of the NRR. As such, the RLMB protocol proposes the use of the dynamic scaling factor *β*_*rd*⁡_. This factor is used as an estimation of the influence of the conditions of the radio channel and it is calculated based on the received hello packets from neighbors.

**Figure 2:**
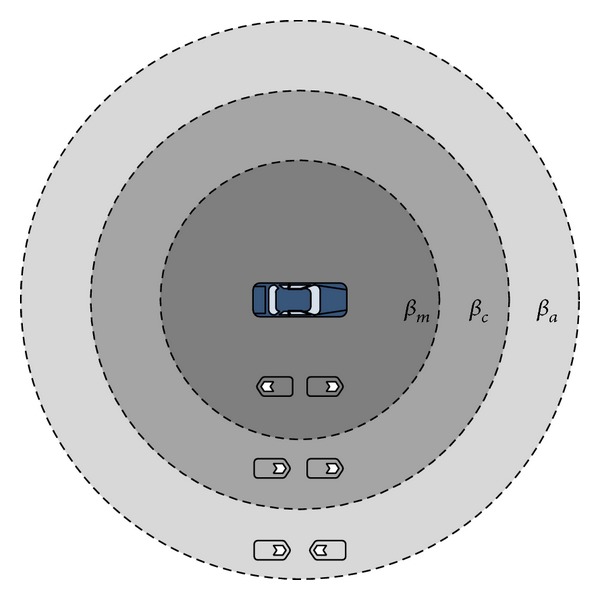
Use of dynamic *β*_*m*_, *β*_*c*_, and *β*_*a*_ factors to determine the maximum allowed transmission range according to the relative direction of vehicles.

Unlike similar BA sender-oriented proposals, in RLMB the assessment is not made for each node. Instead, the link evaluation is made for a particular zone in a cooperative way. As it will be shown in Section 4, this approach allows RLMB making a proper adjustment of the NRR while maintaining a low overhead. Furthermore, according to [25], the relative direction between vehicles has a great impact in the propagation of the signal. Hence, an adjustment factor *β*_*rd*⁡_ of this kind needs to consider the relative direction of vehicles. Thus, RLMB calculates the *β*_*m*_, *β*_*c*_, and *β*_*a*_ adjustment factors, which consider the three possible relative directions taken by two vehicles, as illustrated in Figure 2.

In RLMB the NRR adjustment is performed considering the received power and distance between neighbors. These two aspects are introduced in the RLMB protocol by means of two factors, namely, the *γ* and *δ* factors, which are described next.

The *γ* factor is defined as

(1) $$\begin{array}{c} \gamma =1-\frac{{\text{PL}}_{r}}{{\text{PL}}_{\max}}, \end{array}$$

where PL_max⁡_ is the maximum allowed linear path loss and PL_*r*_ is the current linear path loss. This factor is a measure of how far is the received power from the reception threshold, that is, the minimum acceptable power to detect and decode a packet. A received power close to the reception threshold indicates a poor link quality and *γ* will tend to 0. Conversely, a value far from the reception threshold indicates a better link quality and *γ* will tend to 1.

Note that because of the wireless channel phenomena (e.g., propagation loss and shadowing) a poor link quality is expected for distant nodes. Similarly, a good link quality is expected for the nearby nodes. Thus, if only the *γ* factor is taken into account for the radio adjustment, the *β*_*rd*⁡_ factor would only oscillate between low and high values. In order to address this issue, calculating the *γ* factor considering only the distant nodes seems to be a suitable option at first glance. However, ignoring hello messages from the middle nodes can reduce the efficiency of the protocol because of the reduced availability of information.

To improve the radio adjustment accuracy while maintaining a low overhead, the transmitter-receiver distance is considered in the calculation of the *β*_*rd*⁡_ factor by means of the *δ* factor:

(2) $$\begin{array}{c} \delta =\{\begin{array}{cc} \frac{D_{\left(x,y\right)}}{R}, & \text{if}\;D_{\left(x,y\right)}\leq R\\ 1, & \text{otherwise}, \end{array} \end{array}$$

where *D*_(*x*,*y*)_ is the distance between node *x* and node *y* and *R* is the NRR. This factor is a measure of the distance between neighbors and it is proportional to the NRR.

The value of *β*_*rd*⁡_ must vary according to the channel variations dictated by propagation conditions, so that it can effectively represent the dynamics of vehicular networks. Furthermore, the information available to calculate *β*_*rd*⁡_ may be imprecise because of different factors like a noisy GPS measure or a wrong power measurement at the PHY layer when receiving a hello message. Hence, using a closed expression to calculate the radio adjustment factor *β*_*rd*⁡_ could restrict the protocol accuracy to very specific scenario/channel conditions.

In order to provide RLMB with an adaptable decision making mechanism capable of achieving a satisfactory performance, a smart and adaptive solution for the NRR adjustment based on fuzzy logic [26] is implemented in RLMB. Fuzzy logic has been used in the context of VANETs [19, 26] to make intelligent and adaptive decisions. However, to the best of the authors' knowledge, the fuzzy logic based algorithm presented in this paper is the first to consider the inherent constrains of highway scenarios to dynamically adjust the NRR with a low dependence of the HMR.

#### 3.1.1. Design of the Decision Making System Using Fuzzy Logic

When receiving a hello message at PHY layer, each node calculates the *γ* factor with expression (1). This factor is included in a header of the packet before sending it towards the network layer. In the network layer the *δ* factor is calculated from the received information in the hello packet. Then, both *γ* and *δ* are used as inputs of the proposed fuzzy inference system (FIS) in order to calculate the dynamic adjustment factor *β*_*rd*⁡_.

The proposed decision-making algorithm contains two phases.

**Figure 3:**
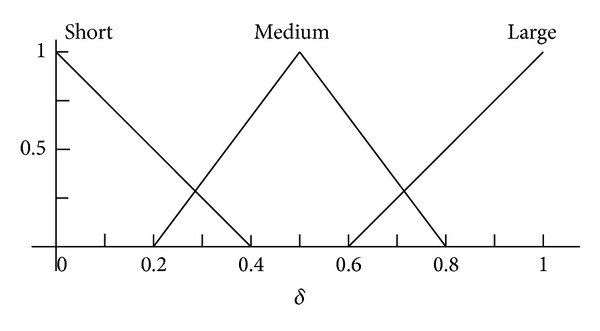
Fuzzy membership function for input *δ* factor.

**Figure 4:**
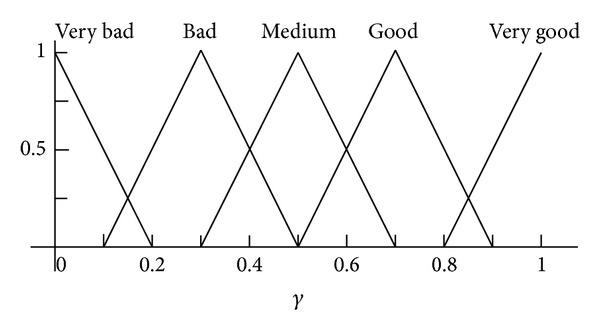
Fuzzy membership function for input *γ* factor.

**Figure 5:**
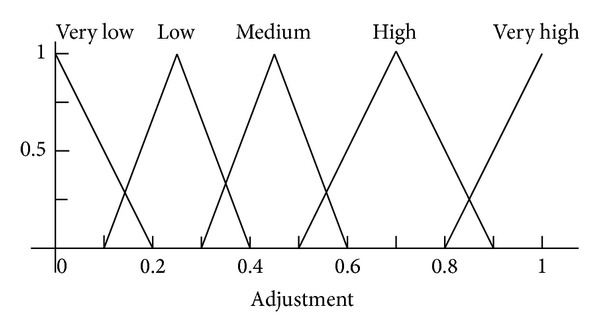
Fuzzy membership function for output variable.

*Phase I.* The initial set up phase when the FIS is formed from two sets of data:

- the construction of the fuzzy memberships based on the individual linguistic values of distances, losses, and adjustments as shown in Figures 3, 4, and 5;
- the if/then rules shown in Table 1. These rules specify the actual combination of these values to properly map the fuzzy values of the distance and losses, to the radio range adjustment fuzzy values.

**Figure 6:**
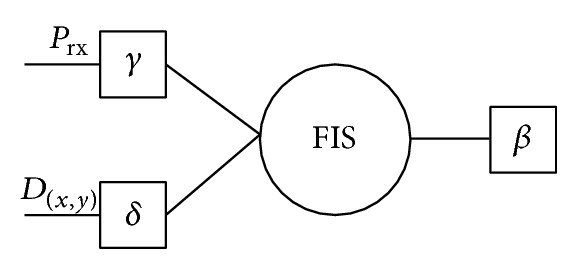
The decision-making system designed to update the adjustment factors.

*Phase II.* The decision-making algorithm (see Figure 6), which is invoked at each hello message arrival and it is fed with the values of distance and losses calculated from the hello message. The decision-making algorithm executes the following main steps:

- mapping the values of the *γ* and *δ* factors to a fuzzy value of *β*_*rd*⁡_ through the FIS;
- using a defuzzification method in order to map from fuzzy value to a crisp value.

To cope with highly transient values at the output of the FIS due to shorter than expected path losses, a moving average is used to smooth such undesired behaviors. The moving average is defined by

(3) $$\begin{array}{c} {\beta_{\mathrm{rd}}}_{n}={\beta_{\mathrm{rd}}}_{i}+\epsilon *\left({\beta_{\mathrm{rd}}}_{\text{current}}-{\beta_{\mathrm{rd}}}_{i}\right), \end{array}$$

where *β*_*rd*⁡__*n*_ is the new radio range adjustment factor in the *rd*⁡ direction; *β*_*rd*⁡_ _current_ is the value of the current *β* in the *rd*⁡ direction; and *β*_*rd*⁡__*i*_ is the inferred value of radio range adjustment in the *rd*⁡ direction. The value of *ϵ* defines the weight of the accumulated evaluation. Because of the high dynamic nature of the VANETs, a higher weight for the current value is desired. Thus, in this paper a value of 0.4 is chosen for *ϵ* in expression (3).

### 3.2. Position Prediction Algorithm

When a vehicle needs to send or relay a packet, the position prediction (PP) algorithm must fill the gap between the last values stored in the neighborhood table and the most updated values. Thus, when the set of relay nodes has to be selected, the PP algorithm is invoked before performing the selection. The PP algorithm implemented in RLMB works as follows.

(1) The position of all neighbors is updated in the neighborhood table by means of
  (4) $$\begin{array}{c} P_{e}=P_{c}+\left({\hat{v}}_{i}*dt+\frac{{\text{acc}}_{i}}{2}*{dt}^{2}\right), \end{array}$$
  where ${\hat{v}}_{i}$ is the last stored velocity vector of the neighbor *i*; acc_*i*_ is the last stored acceleration vector of the neighbor *i*; and *dt* is the information dwell time of the neighbor *i* in the one-hop neighbors table.
(2) The number of entries in the neighborhood table is updated. If the estimated distance for one particular neighbor is larger than the NRR, then this particular neighbor is deleted from the one-hop neighborhood table.
(3) The updated neighborhood table is passed to the relay set selection algorithm.

### 3.3. Relay Set Selection Method

Two classes of nodes are considered in the RLMB protocol: source nodes and relay nodes. The source nodes are vehicles with data to send (e.g., a warning message), while relay nodes are vehicles within the ZOR that must relay the original packet. If a node (source or relay) wants to disseminate a packet, before broadcasting the packet, a set of relay nodes is selected as follows.

(1) Using the respective *β* factor, a threshold for the maximum allowed distance is set for each relative direction *rd*⁡ with expression
  (5) $$\begin{array}{c} {\text{Th}}_{\mathrm{rd}}=\beta_{\mathrm{rd}}*R, \end{array}$$
  where Th_*rd*⁡_ is the threshold for the relative direction *rd*⁡; *β*_*rd*⁡_ is the adjustment radio range factor for the *rd*⁡ direction; and *R* is the NRR.
(2) Neighbors whose distance is below the corresponding threshold, Th_*rd*⁡_, are grouped in three different sets based on their relative direction to the current relay. Specifically, vehicles traveling in opposite directions and moving away belong to group *V*_*m*_; vehicles traveling in opposite directions and approaching belong to group *V*_*a*_; and vehicles traveling in the same direction belong to group *V*_*c*_.
(3) Finally the set of relay nodes is defined considering the following.
  (i) If the current node is a relay node, the next relay node is the farthest node among the vehicles in the *V*_*m*_, *V*_*a*_, and *V*_*c*_ groups in the message propagation direction.
  (ii) If the current node is the source node or if it is located at an intersection, a set of relay nodes must be selected. This set is integrated by the farthest node among the vehicles in the *V*_*m*_, *V*_*a*_, and *V*_*c*_ groups in each direction of the ZOR.

### 3.4. Implicit Acknowledgment Mechanism

As previously mentioned, the aim of the RLMB selection mechanism is to reduce the drop of broadcast packets by considering the constraints imposed by the radio channel and the dynamics of vehicles in highway scenarios. Nevertheless, sometimes the packets may not reach the intended primary relay node because of larger than expected path losses or inaccurate position predictions made by the PP algorithm. Therefore, in order to increase its reliability, the RLMB algorithm implements a basic implicit acknowledgment mechanism. If the current relay does not overhear the sent packet after an iACK time, then the relay retransmits the original packet. Here, the iACK is determined for each node with expression

(6) $$\begin{array}{c} \text{iACK}=\tau +X_{a,b}, \end{array}$$

where *τ* is a constant value and *X*_*a*,*b*_ is a uniform distributed random variable between *a* and *b*.

## 4. Performance Evaluation

In this section, the performance of the proposed RLMB protocol is evaluated. The V2V highway scenario was simulated using the OPNET Modeler simulator [27]. The simulated scenario considered two lanes for cars travelling in one direction and other two lanes for cars travelling in the opposite direction. The length of each lane was set to 3 km and the width to 4 m. In the simulation when a vehicle reaches the end of the road, it is reinserted in the lane with vehicles traveling in the opposite direction. The maximum allowed speed was set to 40 m/s. The radio channel propagation model introduced in [25] for V2V highway scenarios was considered in the simulation setup. The mobility pattern for each vehicle was generated following the intelligent driver model introduced in [28]. This is a popular model used to generate mobility patterns for highway scenarios (e.g., [29, 30]). Additionally, free flow, medium, high, and jam vehicles densities [31] were also considered to evaluate its effects in the achieved performance of the MB protocols.

**Figure 7:**
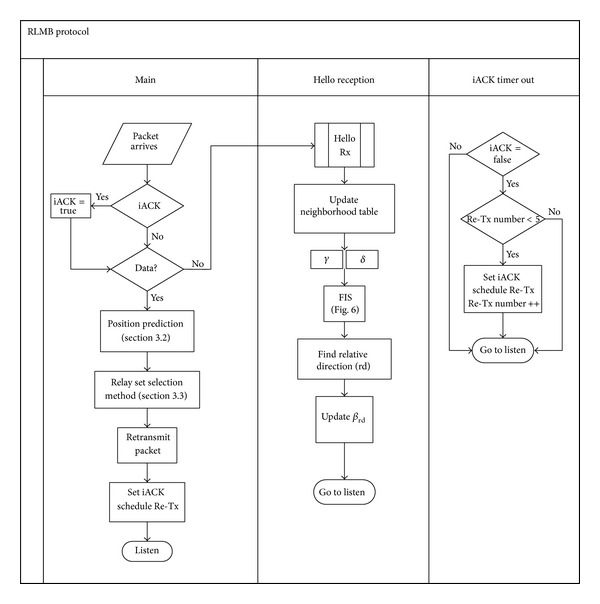
Flowchart of the main processes of RLMB broadcast protocol.

The FUZZBR and the proposed RLMB protocols were evaluated using the previously described scenario. The FUZZBR protocol was used for benchmarking purposes as it is the leading BA sender-oriented protocol in the literature. In fact, in [19] several performance metrics such as delay and packet dissemination ratio were provided for FUZZBR, showing that in a well-connected network this protocol outperforms EMPR and two well-known beaconless MB protocols. FUZZBR was implemented in the simulation testbed following the guidelines provided in [19]. In the case of the RLMB protocol, Figure 7 details the flowchart considered for its implementation.

The performance evaluation was realized in terms of the following performance metrics.

*(a) Packet Delivery Ratio (PDR)*. This metric measures the ratio between the number of vehicles that receive a broadcast packet and the number of vehicles in the ZOR times the packets transmitted by the source. We only consider the first received broadcast to determine the PDR.

*(b) Average End-to-End Delay (EED)*. The EED is calculated as the average delay from the source to each vehicle within a particular segment of the highway. For EED calculation, the highway length was divided in equally spaced segments of NRR width called milestones.

*(c) Retransmissions*. This metric is measured in terms of the number of retransmissions per packets made by each protocol during the simulation.

The aforementioned metrics were obtained considering a crash scenario where a source transmits a warning message with a data rate of 10 Kbytes/s from one edge of the ZOR. A specific propagation distance or a specific time can define the ZOR. For this paper, the entire highway was considered as the ZOR and all vehicles within it were considered as intended recipients. At the beginning of every simulation trial, every transmitter was set to wait for 20 s before starting any data transmission. This 20 s period was set to better allow the exchange of hello messages. In order to reach a stable state, a minimum of 100 trials were performed for each vehicle density, *λ*. The values of each variable used in the simulation are presented in Table 2.

The PHY/MAC layers of the IEEE 802.11p standard were implemented in our simulation, taking as a starting point the IEEE 802.11a project available in OPNET Modeler. The necessary adaptations were performed so that all PHY/MAC settings and parameters correspond to those found in the IEEE 802.11p standard. This approach was previously used in [24, 32] for the evaluation of AODV and DSR routing protocols in VANETs equipped with IEEE 802.11p transceivers. Furthermore, the IEEE 802.11p DCF parameters corresponding to the best effort traffic over service channels (see [33]) were used to modify the IEEE 802.11a OPNET model. Therefore, the minimum and maximum contention windows sizes and the time slot length were adjusted to IEEE 802.11p best effort traffic values. Similarly, the DIFS value was replaced by the corresponding AIFS value. Regarding the PHY layer adaptation, the bandwidth and operating frequency of the IEEE 802.11a OPNET model were adjusted to 10 MHz and 5.880 GHz, respectively, as defined by the IEEE 802.11p standard. For the rest of this paper the modified model will be referred to as adapted IEEE 802.11a/p model.

### 4.1. Numerical Results

**Figure 8:**
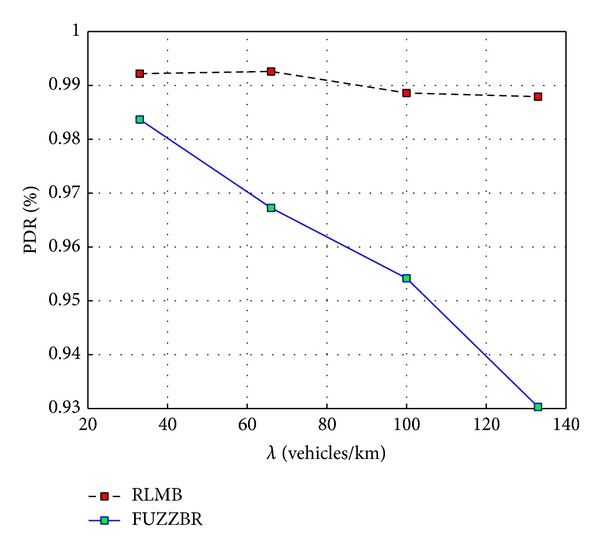
Packet delivery ratio (PDR) obtained with RLMB (0.25 hps) and FUZZBR (1 hps) when increasing the vehicles density, *λ*.

Figure 8 presents graphically the PDR that each protocol is able to achieve versus the vehicles density, *λ*, in the scenario. For this plot a fixed rate of 1 hello messages per second (hps) was set for FUZZBR, while a rate of 0.25 hps was set for RLMB. As shown in this figure, FUZZBR exhibits a poor performance compared to RLMB, especially under higher vehicles densities. This is caused by the larger overhead introduced by FUZZBR when using a rate of 1 hps. Thus, when evaluating FUZZBR in higher vehicles densities, the probability that the retransmission does not reach the intended relay node is increased because of the increment in the number of collisions. In contrast, RLMB provides a stable PDR regardless of the *λ* value. In fact, a drop of less than 1% is shown for the RLMB protocol in higher densities. RLMB exhibits this behavior because the zone-based link quality assessment depends on a much lower degree on the hello messages rate (HMR). Thus, a lower overhead is introduced with the consequent decrement in the number of collisions.

**Figure 9:**
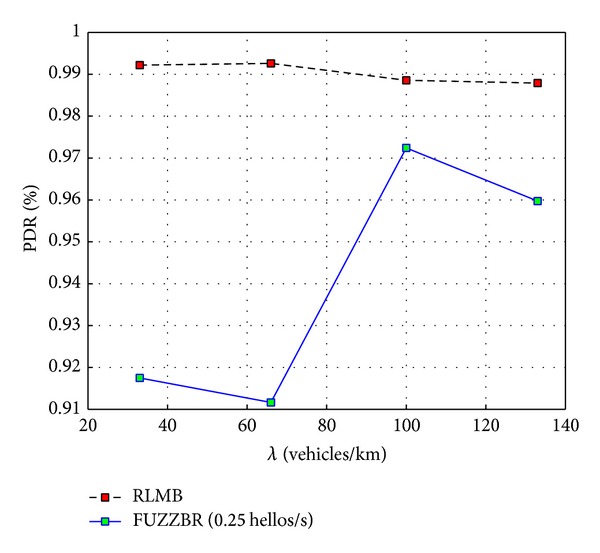
Packet delivery ratio (PDR) obtained with RLMB (0.25 hps) and FUZZBR (0.25 hps) when increasing the vehicles density, *λ*.

Figure 9 shows the PDR achieved when the rate of hello messages per second was set to 0.25 hps for FUZZBR. Thus, FUZZBR and RLMB had the same HMR for this plot. It can be seen in Figure 9 that using a low HMR in FUZZBR improves its performance for high vehicles densities. In high vehicles densities the dynamic of vehicles is lower. Thus, reducing the HMR along with the low dynamic of vehicles enables a performance improvement for FUZZBR in these cases (even though there are a major number of vehicles sharing the channel). However, the suitability evaluation for the selection of the relay nodes in FUZZBR is closely related to the HMR. Thus, FUZZBR has an important performance drop when the dynamic of vehicles increases, as observed in Figure 9 for the first two values of *λ*.

Despite the FUZZBR improvement observed in Figure 9 for high vehicles densities, it can be readily seen that RLMB still provided a better PDR than FUZZBR for all densities. Therefore, RLMB exhibited a better PDR when compared to the one achieved by the benchmark protocol for both low and high HMRs.

**Figure 10:**
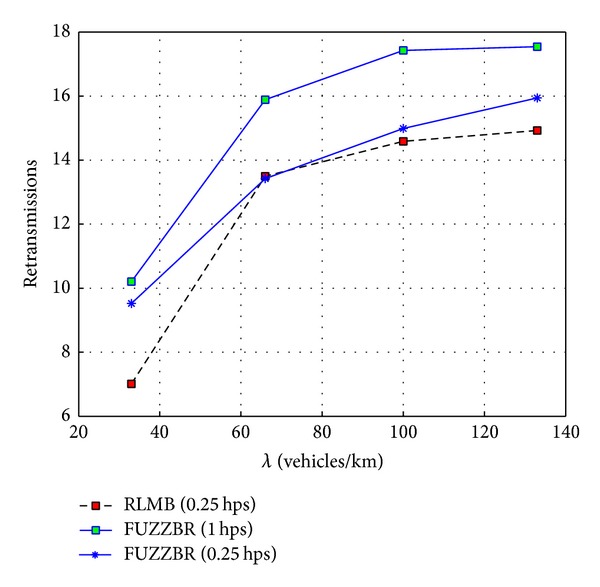
Number of retransmissions obtained for RLMB with 0.25 hps compared with the retransmissions obtained for two HMR (0.25 hps, 1 hps) of FUZZBR when increasing the vehicles density, *λ*.

Figure 10 illustrates the effects of increasing the vehicles density on the average number of retransmissions. Both protocols need a higher number of retransmissions when *λ* increases. The reason for this is because with the increment in the number of vehicles per km, the number of nodes sharing the channel increases. Thus, there is an increment in the probability of collision, which may cause that a packet does not reach the next relay or that the implicit acknowledgment does not reach the previous relay. However, because of the lower HMR, RLMB introduces less overhead. Thus, the retransmissions needed for the different densities are always lower than those needed in FUZZBR. Additionally, note that even when the HMR of FUZZBR is reduced, RLMB provides a better performance. This is the effect of the proposed PP algorithm, which handles the dynamic of vehicles efficiently.

**Figure 11:**
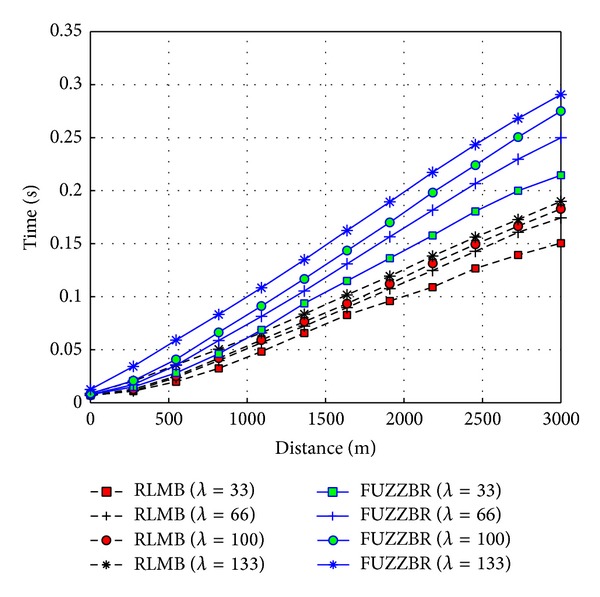
EED obtained with RLMB (0.25 hps) and FUZZBR (1 hps) when increasing the transmitter-receiver distance for different vehicles densities, *λ*.

Figure 11 graphically presents the effects of increasing the source-destination distance for different vehicle densities on the EED. Note in this figure that the packets disseminated by RLMB reach the end of the ZOR in a lower time for the different values of *λ*. This is the result of two aspects: (1) RLMB requires a lower number of retransmissions; and (2) the introduced overhead related to the hello messages is also lower in RLMB than in FUZZBR.

**Figure 12:**
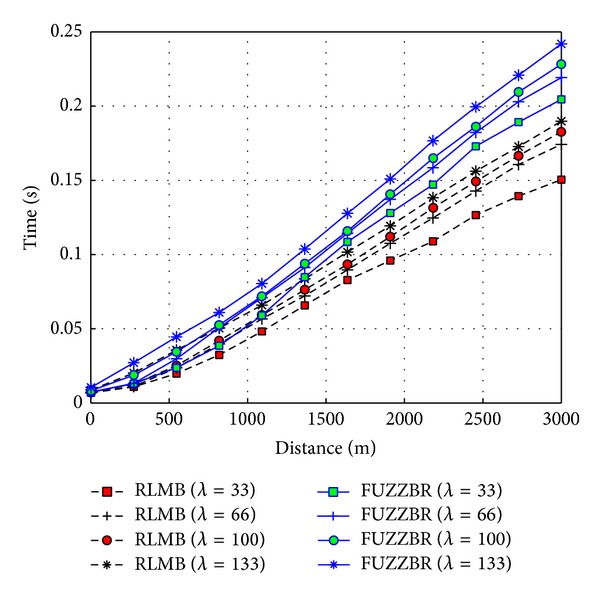
EED obtained with RLMB (0.25 hps) and FUZZBR (0.25 hps) when increasing the transmitter-receiver distance for different vehicles densities, *λ*.

When disseminating the packet along the ZOR, fewer delays are introduced by RLMB compared to FUZZBR.  Figure 12 shows a comparison of the EED obtained for RLMB with the EED obtained for FUZZBR with a HMR that matches the one used by RLMB. Under these conditions, FUZZBR achieves better performance for EED when compared to those obtained for FUZZBR with high HMR. However, the evaluation of the vehicles mobility performed by FUZZBR is closely related to the HMR. Thus, a low HMR causes a higher number of errors in the next relay selection method of FUZZBR. Hence, a higher number of retransmissions are needed in comparison with RLMB. Therefore, RLMB outperforms FUZZBR in both scenarios as it can be seen in Figure 12.

## 5. Conclusions and Future Work

In this paper a new low-overhead and reliable multihop broadcast protocol for VANETs called RLMB is introduced. The RLMB protocol proposes a point-to-zone computation of the link quality instead to a point-to-point assessment. The design of RLMB was aimed to address the two trade-offs arising in BA multihop broadcast protocols deployed in highway scenarios: the trade-off between reliability and redundant broadcast and the trade-off between reliability and the number of hello messages needed. As such, the performance of RLMB was compared with the performance shown by the FUZZBR protocol, which is one of the few broadcast protocols that consider both the dynamic of vehicles and the radio channel in the selection of the set of relay nodes. These contribution findings indicate that for low vehicle densities in highway scenarios, RLMB provides a higher PDR with a lower number of retransmissions compared to the results obtained when using the leading protocol FUZZBR. This is achieved by means of the PP algorithm altogether with the proposed radio adjustment of RLMB. Furthermore, the average EED is lower as well in RLMB than in FUZZBR for this case. In the case of high vehicles density scenarios in highways (which is likely to be the most relevant crash scenario), the proposed RLMB protocol also outperforms FUZZBR. The reason for this is because the point-to-zone link quality assessment reduces the dependence of RLMB on the HMR. Therefore, because of the reduced HMR and the lower number of retransmissions needed, RLMB introduces less overhead than FUZZBR. This feature of RLMB also helps in reducing the number of collisions. Additionally, the PP algorithm and the adaptive *β*_*rd*⁡_ factor help to perform better relay set selections in highway scenarios. This can be drawn from the fact that a lower number of retransmissions are generated when using RLMB compared to FUZZBR.

With the results presented in this paper, it has been shown that, with a point-to-zone link quality assessment, the dependence of the broadcast protocol from the HMR is reduced. Therefore, an improved performance for different vehicle densities can be achieved. In that sense, it can be stated that in a well-connected vehicular network RLMB efficiently handles the reliability/overhead trade-off. Furthermore, RLMB outperforms the leading BA sender-oriented protocol for multihop broadcast in connected highway scenarios.

In this paper a fixed HMR is used for RLMB. Thus, future work includes developing an adaptive HMR mechanism based on the current network conditions in order to make RLMB suitable for urban scenarios. Additionally, the implemented FIS was designed based on the overall performance in different network scenarios. The RLMB protocol performance can be improved if the FIS can be adapted to specific scenario conditions. Therefore, future work will include optimizing the FIS for different deployment scenarios such as a disconnected VANET.

## Figures and Tables

**Table 1:** Knowledge structure based on fuzzy rules.

If	&	If	Then
*δ*		*γ*	Adjustment
Short		Very bad	Very low
Short		Bad	Very low
Short		Medium	Medium
Short		Good	High
Short		Very good	High
Medium		Very bad	Low
Medium		Bad	Low
Medium		Medium	Medium
Medium		Good	High
Medium		Very good	Very high
Large		Very bad	Low
Large		Bad	Medium
Large		Medium	High
Large		Good	Very high
Large		Very good	Very high

**Table 2:** Values used for each variable in the simulation scenario.

Scenario parameter	
Maximum velocity	40 m/s
Vehicles density, *λ*,	[33, 66, 100, 133] v/km
Highway length	3 km
Packet size	1 Kbyte
Base frequency	5.880 GHz
Data rate	6 Mbps
Transmission range	250 m

FIS parameters	

Combination method	Min-max
Defuzzification Method	Centroid of area
